# Unpacking the implementation blackbox using 'actor interface analysis': how did actor relations and practices of power influence delivery of a free entitlement health policy in India?

**DOI:** 10.1093/heapol/czaa125

**Published:** 2020-11-06

**Authors:** Rakesh Parashar, Nilesh Gawde, Anadi Gupt, Lucy Gilson

**Affiliations:** 1 School of Health Systems Studies, Tata Institute of Social Sciences, Mumbai , India and Health Systems, Oxford Policy Management Limited, India; 2 School of Health Systems Studies, Tata Institute of Social Sciences, Mumbai, India; 3 National Health Mission, Government of Himachal Pradesh, Shimla, India; 4 Division of Health Policy and Systems, School of Public Health and Family Medicine, University of Cape Town, South Africa and Department of Global Health and Development, London School of Hygiene and Tropical Medicine, London, UK

**Keywords:** Health policy, health services research, health systems research, implementation, power, policy implementation, policy analysis, framework, qualitative research, policy process

## Abstract

Exploring the implementation blackbox from a perspective that considers embedded practices of power is critical to understand the policy process. However, the literature is scarce on this subject. To address the paucity of explicit analyses of everyday politics and power in health policy implementation, this article presents the experience of implementing a flagship health policy in India. Janani Shishu Suraksha Karyakram (JSSK), launched in the year 2011, has not been able to fully deliver its promises of providing free maternal and child health services in public hospitals. To examine how power practices, influence implementation, we undertook a qualitative analysis of JSSK implementation in one state of India. We drew on an actor-oriented perspective of development and used ‘actor interface analysis’ to guide the study design and analysis. Data collection included in-depth interviews of implementing actors and JSSK service recipients, document review and observations of actor interactions. A framework analysis method was used for analysing data, and the framework used was founded on the constructs of actor lifeworlds, which help understand the often neglected and lived realities of policy actors. The findings illustrate that implementation was both strengthened and constrained by practices of power at various interface encounters. The implementation decisions and actions were influenced by power struggles such as domination, control, resistance, contestation, facilitation and collaboration. Such practices were rooted in: Social and organizational power relationships like organizational hierarchies and social positions; personal concerns or characteristics like interests, attitudes and previous experiences and the worldviews of actors constructed by social and ideological paradigms like their values and beliefs. Application of ‘actor interface analysis’ and further nuancing of the concept of ‘actor lifeworlds’ to understand the origin of practices of power can be useful for understanding the influence of everyday power and politics on the policy process.

**KEY MESSAGES**

- This analysis illustrates that health policy implementation is not merely a function of framing implementation procedures but is significantly influenced by the actor relationships and power struggles, which are rooted in the lived experiences of actors.
- Actor interface analysis provides an entry point to locate practices of power in interface encounters. It can help understanding of the nature of power practices as well as their influence on health policy implementation.
- *Actor lifeworlds* as a concept considers the often neglected and lived realities of policy actors manifesting in everyday politics and power practices. These include—power relationships based in organizational and social positions; personal characteristics such as interests, attitudes and experiences and socially constructed worldviews and ideologies of actors.

## Introduction

Implementation of health policies is the blackbox that holds many answers to the questions of how policies can meet their intent and how intended users of the policy see them in practice. Implementation has been widely recognized as a central area for health policy analysis (Gilson *et al.*, 2018) and has been one of the drivers shaping the field of health policy and systems research (Bennett *et al.*, 2011, 2018). Implementation is recognized as a critical phase in the policy process and is one of the four phases in policy cycle according to the ‘stages heuristics’ model (Laswell, 1956; Brewer and DeLeon, 1983; Weible *et al.*, 2012). Broadly, all policy processes are contingent on wider socio-political contexts and are shaped by the interaction of involved actors, their knowledge and power dynamics, as well as by aspects of decision making and the policies in question (Walt and Gilson, 1994). A people-oriented approach that considers human agency and attributes at the centre of health systems and health policy has, thus, been recognized as important in transforming the practice of health systems and policy (Sheikh *et al.*, 2014). Power is at the heart of every policy process (Erasmus and Gilson, 2008; Gilson and Raphaely, 2008; Lee, 2015). Health policy actors use their power and agency to engage in contestation, negotiation and collaboration, giving real-life direction to the policy process. Yet few studies purposefully and fully examine the practices of power applied by policy actors and how they shape the implementation. Three recent reviews—a review of discretionary power by frontline workers and managers, the health policy analysis reader and ten best resources on power, have highlighted the need for more theoretically diverse studies that are focused specifically on the local practice knowledge, around the values and meanings that influence these micro-practices of power (Gilson *et al.*, 2014, 2018; Sriram *et al.*, 2018). Moreover, the implementation of health policies has been less commonly studied for understanding the influence of actors’ agency and power, their interactions and relationships and how actors assemble the surrounding structures and contexts in LMICs (Sheikh and Porter, 2010; Lehmann and Gilson, 2013; Sheikh *et al.*, 2014; Barasa *et al.*, 2016) and respond to implementation needs.

Some of the theoretical domains used in the broader policy implementation literature which draw both from top down (Pressman and Wildavsky, 1973; Hogwood and Gunn, 1984) and bottom up (Lipsky, 1980; Barrett and Fudge, 1981; Malik *et al.*, 2014) views of implementation (Hupe and Hill, 2016) can arguably enrich the field. This could be achieved by not only considering implementation processes and contexts (Walt and Gilson, 1994) but also examining agency, decisions, actions and power dynamics of actors as informed by their beliefs, values, interests, motives and norms (Gilson *et al.*, 2011; Hudson and Lefttwich, 2014; Sheikh *et al.*, 2014). Development sociologist, Normal Long in his work on actor-oriented perspectives of development interventions (Long, 2001), has illustrated how the lived experiences of policy actors, their interactions and power struggles, shape policy implementation in a way that it is practised differently from its original blueprint (Long, 2001; Long and Liu, 2009). This paper attempts to contribute in this direction. It presents an implementation analysis of one of the flagship policies in maternal and child health in India, using an actor-oriented perspective (Long, 1989). The paper illustrates how power struggles of actors influenced the implementation of this policy using actor interface analysis (Long, 2001).

We studied the implementation of Janani Shishu Suraksha Karyakram (JSSK), which translates to ‘mother and child safety scheme’. JSSK, a scheme launched by the government of India, promised completely ‘free’ maternal and child health services in public hospitals in India in the year 2011. However, JSSK service users still spend money out of their pockets [Government of India, 2014; Tyagi *et al.*, 2016; Indian Institute for Population Sciences (IIPS) and ICF, 2017]. The reasons behind these continued expenses have not been discussed, most of which could be related to implementation fallacies (Sabatier, 1986).

Trying to understand this gap between the policy promise and policy practice, our broader study analysed the overall influences on implementation of JSSK. This paper specifically presents the role of actor relationships and power in the implementation of JSSK using actor interface analysis (Long, 1989, 2001).

## Methods

Considering that there could be multiple coexisting possibilities that would affect the implementation course of JSSK, we used a flexible, qualitative study design (Robson, 2009) to approach this inquiry. To get into the depths of this issue and build rich insights about implementation, we narrowed down the study geography to one Indian state as a case (Yin, 1999) of JSSK implementation. We chose the state of Himachal Pradesh (HP), as HP was one of the first states to adopt JSSK. But, the state incurred higher than the national average of out of pocket expenses on free services promised under JSSK, after more than 5 years of implementation (Government of India, 2014; IIPS, 2017). The approach to data collection, analysis and use of theory was reflectively considered based on the first round of fieldwork, as we noticed actor relationships and power dynamics emerging as one of the dominant features of JSSK implementation. We drew upon the framework analysis approach (Ritchie and Spencer, 1994) and developed a framework for analysis, based on the categorization of ‘*actor lifeworlds*’ (Table 1). This categorization was informed by the ‘elements of actor interface analysis’ (Long, 2001; Asian Productivity Organization, 2003). For this study, we considered practices of power as acts of influencing decisions or actions of actors and observed power as a diffused concept used in the Foucauldian idea (Gordon and Marshall, 1980.) The acts of influencing each other’s actions were guided by the power struggles at the actor interfaces and drawing on elements of actor interface analysis, we included practices of domination, control, resistance, contestation, collaboration and negotiation as practices of power (Long, 2001).

**Table 1:** *Actor lifeworlds*: a framework for contributing reasons for practices of power in actor interfaces formed in a policy process

Broad categories of *lifeworlds*	Relationships of power	Personal life concerns or characteristics	Social/cultural/ideological standing or worldviews
Contributory elements for each category	Organizational/ hierarchy and professional autonomy, resourcefulness; social positions or status, relations of gender, caste, class and professional expertise	Individual interests, motivation, attitude, identity, image, recognition, professional training, previous experiences, personal commitments, energy, cognitive and behavioural traits	Values, beliefs, ideologies, moral and ethical positions, organizational and cultural norms and patterns

Adapted from Long (2001).

We illustrate below, how actor interface analysis was operationalized in this study.

### Steps in conducting actor interface analysis

As a first step, we developed a guiding framework, as shown in Table 1, to guide the analysis of practices of power. This framework uses the constructs of routine social realities of actors, called ‘*actor lifeworlds*’ which are generally neglected in policy interventions and which create a basis of engagement of policy actors in actor interfaces (Long, 2001). Lifeworld term was used by Schutz (1962) ‘*to depict the “lived-in” and “taken-for-granted” world of the social actors*’ and was later elaborated upon by Schutz and Luckmann, (1973) . These social realities or lived experiences of actors could be based in their more visible relations of power such as social and organizational positions; can be a construct of actors’ personal characteristics or concerns or could be related to worldviews informed by actors’ social, cultural or ideological standings. Each of these categories could have multiple contributing characteristics, as depicted in our framework of *actor lifeworlds* in Table 1. However, it is worthwhile to clarify here that these categories and elements within these categories are not mutually exclusive, overlap in some constructs and influence each other.

As a next step, we tailored our data collection to capture not only the practices of power but also the lived experiences of actors guided by *actor lifeworlds* framework. The data collection included iterative in-depth interviews (IDIs), document reviews and observations of actor interactions. IDIs covered a range of implementing actors from state and district levels, including technocrats and bureaucrats, managers, service providers and JSSK beneficiaries (Table 2). The questions included in IDIs focused on JSSK related experiences of participants and enquired in detail about participants’ interactions with other actors in relation to implementation decisions and as well as their interests, experiences, beliefs, relationships, etc. (see Supplementary file S1).

**Table 2:** In depth interviews

Participants category	IDIs	Characteristics included
Care givers of JSSK beneficiaries	8	Maternity and infant wards; spent money/did not spend money; levels of health facilities
Staff nurses	7	Labour, neonatal and infant care units; contractual and regular staff; early career, mid and late in career stages
Birth attendants (Dai)	2	Near retirement
Administrative nurses	3	Mid-career to near retirement; Matron and Ward in-charges; level of health facilities
Health facility in-charges	4	Medical college hospital, district hospital and subdistrict hospitals
Store in charge	2	Management committee member, store in charge, purchase committee member
Practicing clinicians	8	Obstetricians, Paediatricians, Radiologists, non-specialist doctors from different level of facilities
Block medical officers	5	Also served as facility in-charges
District level officers	4	District managers and programme nodal persons
State level officers	8	Programme managers and officers (including state directors, bureaucrats)
Total IDIs	51	About 22 h of recording and 8 h of non-recorded (summarized) interviews

As shown in Table 3, we observed interactions of service providers with each other and with JSSK clients in health facilities, observed interactions of managers in state and district offices and attended official meetings to note interaction patterns and decision-making processes. The documents available in the public domain were accessed, and specific letters were accessed from offices on request. The JSSK guidelines, 15 state letters and four media reports were reviewed.

**Table 3:** Observations and documents reviewed

Observations (nonparticipant)		
Observed sites	Hours	Observation focus
PNC Ward, SNCU, review meetings (state, district), state level training, offices (NHM, facility in charge, matron)	27	Setting, work atmosphere, interaction patterns (tensions, negotiations, collaborations, etc.), any JSSK relevant events (service delivery, procedures, demands, response, grievances, resolution, etc.)
Documents collected		
JSSK guidelines (national, state)		
Letters and office correspondence on JSSK—NHM and HP Govt website		
Some letters related to specific information from state NHM office—accessed on request		

We transcribed the interviews and made summary notes from observational data and documents. We used a software, ‘Dedoose for qualitative analysis’, version 8.2.14 for coding, organizing and linking data across files. Coding was done by the first author and supported by a research consultant. The consultant first coded a few transcripts with the first author and independently coded other transcripts based on this learning. Some files were independently coded by a field expert for validity purposes. The codes and themes were later discussed together to arrive on a consensus. We first coded the transcribed data to map the procedures which were laid down for JSSK. As the next step in coding, we interpretively identified actor interfaces formed during implementation course by applying ‘type of interface’ codes to actor interactions and sub-coded practices of power into the acts of collaboration, control, domination, resistance, negotiation and contestations. The next significant step in this process was to examine lived experiences of actors, which we coded into three broad categories of ‘*actor lifeworlds*’. Within each lifeworld category, we sub-coded the contributory elements, as shown in Table 1. The effects of power practices were organized in two broad categories*—*strengthening of implementation (helping the delivery of policy promises) and contrasting the implementation (thinning of policy intent).

Ethical approval for this study was obtained from the author’s institute.

## Results

### JSSK implementation journey and main challenges faced

HP was one of the early adopters of JSSK as the state chief minister announced JSSK entitlements soon after JSSK guidelines were available. JSSK rollout was initiated with an official letter and emphasized on ‘assuring nil out of pocket expenses’ from intended beneficiaries of JSSK in all government health facilities in the state. To make the promised services available for free in all public hospitals, the state framed new procedures and guidelines for the provision of these services in all public facilities. For example, to make the required medicines available in health facilities, a new procurement process was laid down based on the list of medicines and consumables, which was based on JSSK guidelines from the centre. This medicine list and procurement guidelines were updated later at many points. Similarly, for diagnostic tests, all user charges for the tests available within the hospital were removed, but all the laboratory and radiology services required under JSSK were not available in most facilities. After a couple of years, laboratory services in public hospitals were outsourced to a private company, which made most diagnostic tests available in the hospitals and charges for these were exempted for JSSK beneficiaries. For ensuring free availability of ultrasonography services to all clients, private tie-ups were attempted with occasional success. Likewise, for free transport services, ambulances in health facilities were much less in number compared with requirements of JSSK. Later, the state purchased an outsourced private ambulance service for all emergencies as a centralized ambulance system and a special drop back to home ambulance service was also initiated in the year 2014.

However, with successes in some areas, the full potential of these provisions could not be achieved because of inconsistencies in their delivery even in the better-performing facilities. This often left out many beneficiaries, who still paid money from their pockets, against JSSK promises. Overall, some of the major challenges faced for ensuring free drugs and consumables, diagnostic tests and transport services were:

1. Inadequate availability and inconsistent delivery of items in the JSSK drugs and consumable lists in some districts and health facilities especially in the larger hospitals.
2. Ultrasonography (USG) services remained available only at larger hospitals, which made beneficiaries travel big distances to avail USG services and private USG service providers outside these hospitals bloomed in most places.
3. While transport services were available to many clients, these were also inconsistent, particularly in cases of inter-facility transfers and drop back services to the home.

Findings from the overall enquiry of JSSK implementation suggest that most of these problems had their roots beyond framing implementation guidelines and procedures. These were predominantly influenced by power practices of actors across levels of the health system; the overall context of the state health system; politics; corruption; interaction of JSSK with other policies and programmes and management of JSSK implementation processes including guiding procedures, monitoring, reporting and measurement processes. However, as the focus of this paper remains on practices of power which we discuss below.

### Actor interfaces, practices of power observed

Table 4 outlines some key examples of actor interfaces and practices of power which were identified in this experience and which influenced implementation related decisions and actions. These interfaces were formed in relation to an implementation step or a procedure. The actors involved in JSSK implementation ranged from ministers and senior officers from central government as well as the state directors and managers from the state health department and NHM, district level managers, health facility managers and service providers (doctors, nurses and traditional birth attendants in some cases). The communities themselves were an integral part of implementation by availing JSSK services and at times influenced the decisions and actions of health service providers. Moreover, private service providers, often concentrated outside public hospitals, played an indirect role in shaping the implementation.

**Table 4:** Examples of actor interfaces and practices of power in JSSK implementation

Example of interfaces observed	Practices of power and related implementation issue
Political interfaces (centre-state/politician-managers/managers-private owners)	Centre domination on policy and programmatic agenda over the decision of JSSK rollout in HP
	Resistance and contestations by private service providers against free medicines and tests in public hospitals Negotiations by managers with private providers
Interfaces among middle managers across levels (facility/district/state)	Resistance by facility managers to follow top down instructions on JSSK documentation and reporting Contestation for getting credit about delivering free services among state and district managers State domination over reporting needs
	Collaboration for local problem solving and implementation needs for policy among some managers
	Top down push by state to control implementation steps and guidelines, Resistance and avoidance by facility managers
Interfaces among doctors and managers in health facilities	Resistance of doctors towards a restrictive medicine list; Resistance of doctors for using generic drugs Negotiations and contestations from doctors about need of higher end and more modern medicines citing quality issues
	Resistance related to prescription of ultrasonography to pregnant women
	Resistance from doctors for involvement in national programmes
Interfaces among nurses and managers health facilities	Control of administrators on resources Negotiations by nurses for availability of medicines
	Contestation and negotiation by nurses with doctors on choice of free medicines and tests for patients
Interfaces among beneficiaries and service providers (doctors/nurses/managers)	Doctors facilitation for service delivery to clients
	Domination of doctors and nurses on service delivery decisions (sending a client away)
	Domination of doctors on patient’s choice for medicines or treatment and consent from patients
	Negotiations and contestations of beneficiary and managers for better quality or more advanced services or services bypassing the guidelines
	Doctor and service provider control over providing USG service and client negotiations for USG service access

The practices of power on various types of interfaces are shown in the first column of Table 4, and the second column shows various practices of power in relation to implementation decisions and actions.

### The lifeworlds of actors underpinning the actor interfaces and practices of power

The actor interfaces formed in this experience and the practices of power noted on these interfaces were underpinned by a variety of lifeworld experiences of actors, which are illustrated in Table 5. The lifeworld analysis is divided into three lifeworld categories as guided by Table 1. However, it is important to note that these categories of *lifeworlds* are not mutually exclusive and interact as well as overlap with each other as also mentioned in the ‘Methods’ section. Also, most practices of power could be underpinned by multiple lifeworld experiences as depicted in Table 5 below.

**Table 5:** Type of power practices and contributing *actor lifeworlds*

Types of power practices observed at actor interfaces	Underpinning lifeworld elements		
	Positional power relations	Personal concerns/characteristics	Social, cultural, ideological standpoints
Centre actors’ domination on policy and programmatic agenda	Organizational power and budgetary control of politicians and central actors		
Resistance, contestations and negotiation by private service providers,	Influential social positions and cumulative power of private lobbies	Personal interests of local politicians, managers and doctors in kickbacks	
Resistance to follow top down instructions on JSSK documentation and reporting	Social positions of being junior and senior in profession	Unwillingness and non-cooperating attitude of some managers; need for recognition and credit for managers	
Domination of doctors and nurses on service delivery decisions (sending a client away)	Professional autonomy on clinical decisions of doctors		
Domination of doctors on patient’s choice for medicines or treatment and consent from patients	Professional position, social positions of influence of doctor; Low knowledge of patients		
Negotiations and contestations of beneficiary and managers for better quality	More informed clients and exercising knowledge, use of social influence by patients		Beneficiary belief in patient rights and entitlements
Doctor and facility control over availability of USG services and client negotiations	Organizational and professional (medical) power of doctors	Absence of choice and personal need of patients to avail services from private; financial interest of doctors	Accepted norm for not being accountable to patient needs; Belief in incentivization to doctors as a systems responsibility
Collaboration, facilitation for local problem solving and implantation needs for policy		Commitment, energy, problem solving attitude of one manager	Faith in participatory and collaborative management of a manager
Doctors facilitation for service delivery (all services to a client)			Doctor’s professional ethics and moral sense of duty towards society and poor

#### Positional power relationships of actors

Often visible relationships of power that were embedded in the organizational and social positions of actors influenced how actors interacted with each other, in relation to a policy issue. For example, at an interface of state and central officers related to the decision of JSSK rollout in HP, practices of domination and control by politicians and central actors were observed as seen in the example below:

*Now, what can I do, if my entire programmatic funding is going to come from the Government of India! … …. So we willy-nilly fall in line with the national agenda. That is how…most states have operated. Schemes like JSSK, despite not being our main priority, we must implement them… And we also must keep bringing money to the state, to save our reputations* (Senior officer*—*1).

In another interface encounter related to politics of health service delivery, private lobbies negotiated with mangers, doctors and politicians to ensure that their medicines and tests are purchased by either patients or the governments themselves. The influential power of private lobbies and control on decision making of politicians along with personal financial benefits system actors came together to allow the continuation of this arrangement as seen in below example (Table 5):

*In the beginning, it was a rebellion kind of a situation from private shops. They would tell us, … why are you bringing this free system here, then they were making the local ministers call … that why are you giving medicines from the hospital?* (Middle manager—2).

Beneficiaries held the least power in their position in service delivery chain because of their social positions, lack of knowledge compared with service providers and being dependent on them to avail promised entitlements of JSSK. While in most cases, beneficiaries consented to what was provided, they, less commonly, also exerted pressure on the service providers to avail quality services. Resourcefulness, connections with influential people and politicians and access to better information were the reasons which enabled beneficiaries to demand better services (Table 5).

*Patients, sometimes, don’t want to stay for 48 h after childbirth…. They say that send us back immediately, so we must tell as per guidelines, that if you don’t stay for 48 h, we can’t send you by 102 (Drop back service ambulance). But now, somewhere from above this pressure comes, phones start coming…to drop her back home … send her by the vehicle. So we have to do that* (Nurse—1).

#### Personal concerns or characteristics

We found many interfaces where managers engaged in cooperation and collaboration with each other to make feasible implementation decisions and acted together to solve problems. While some of such collaboration was related to the organizational powers, most of it came from personal characteristics, previous experiences and ideological belief in a participatory and collaborative work environment. As one state manager highlighted:

*No, the difference of opinion is everywhere. You cannot say that everybody thinks the same way, right? But we try to hear everyone and support districts. And like from the state level, we sat and hand-held district teams, I think the districts ones also did the same thing with the health facilities. As a result of which many problems of JSSK, were resolved* (Middle manager—1).

The lifeworld experience of managers, in this case, emanated from an attitude of solving problems (personal characteristics) and a belief in JSSK as a useful policy for communities (individual understanding).

Contrary to this experience, at one interface we noted that facility managers avoided displaying JSSK entitlements and list of medicines available at facilities and many facilities resisted doing it (Table 4). Origin of this practice of resistance from facility managers was based in their personal experience as they had faced pressure from service users earlier when they had displayed another policy benefit but could not provide it for lack of resources. Partly, this was also an organizational power exercise as the state managers, as well as district managers, had to report whether facilities have displayed the entitlements and free medicine lists (Table 5). This was an interface of bottom up resistance and negotiation at the end of district and subdistrict managers, influenced by their previous experiences as well as organizational positions.

*… In reality, we have not been able to give JSSK widespread publicity…The meaning of giving widespread publicity was this that we told them (district and facility managers) that you display the list of free medicines in your hospital. … now if they display and patients demand it … their … accountability … increases …. So obviously … they were … apprehensive …!* (Middle manager*—*1).

From the state manager’s point of view, this was a result of unwillingness and a lacklustre attitude of districts. But the district managers indicated that this was a struggle to resist the top down and authoritative instructions to implement programmes (without consulting them), many of which came informally. The contrasting view of the district managers emanated from previous their previous experiences where the suggestions they offered related to implementation were not considered by the state managers. This led to a feeling of frustration and district officers started avoiding state instructions. Partly, this was also linked to organizational positions of power of state managers and was a personal coping mechanism of district managers to deal with such pressures (Table 5). One subdistrict level manager, who was also a facility in charge (and a clinician) mentioned:

*We used to give suggestions earlier also; we give suggestions even now…And there is another thing that the technical review and decisions are made by the state only, we have no role in improving technical guidance. We are asked only to give implementation solutions and results* (Middle manager—3).

Like power struggles within managers, doctors and managers also had lifeworld differences as well as commonalities. Doctors negotiated for incentives on ultrasonography services as radiologists were a scarce entity, and they would earn much higher if worked in private setups. Similarly, on the provision of prescribing the medicines only from the JSSK list, doctors, especially who worked at bigger hospitals such as medical colleges, resisted sticking to policy guidance. Their individual financial interests and peer comparison with earning more money manifested through these negotiations and resistance (Table 5). One can argue that these are the medical powers of the doctors used for this type of resistance. However, we assigned the personal lifeworld category here as the personal motives, especially the financial interests of doctors appeared to drive this resistance. This resistance was facilitated by their organizational or medical powers, which is an interaction of two lifeworld categories. At times doctors’ professional training and their recognition as an able specialized clinician also mattered. We see these personal characteristics in the examples below:

*They (pregnant women) are giving Rs. 1200, Rs. 2400 to private for all their ultrasounds. I am myself looking for regulation for this. If I was a radiologist, I would have no incentive for doing ultrasound …. even if I do 100 ultrasounds in a day, I will have no incentive. But if I send 100 ultrasounds outside, I will get minimum Rs. 10 000 indirectly. I have been telling this to state, but they don’t listen* (Obstetrician—3).

#### Social, cultural and ideological worldviews

In response to a policy implementation step, actors make meanings of their social, cultural, moral, ethical and ideological standpoints, which guides their actions. On one such experience, a doctor ensured completely free treatment of a delivering mother and her baby. This was linked to professional and moral ethics, a sense of duty towards society and the professional autonomy of doctors on delivering such services (Table 5).

*… I haven’t paid any money in this hospital, and we are here for about 14 days now. My baby was borne by operation, and since then she is admitted here. Everything has been free for us. The doctor who admitted us immediately decided to perform an operation for delivery …. This is my 2nd baby, but after four abortions before this. We are very poor. If, he didn’t help us, God knows what will happen to us* (Beneficiary*—*1).

*This did not only seem to be a medical emergency to me, but I realised that the patient was very poor, and her husband couldn’t afford anything by himself. It was a premature delivery, and I thought it was my duty to provide them with everything which was available to us. If the government is providing free services, why should we bother about anything* (Obstetrician*—*1).

In a different experience, professionally accepted practice of prescribing branded medicines and organizational norms of not being accountable to patients and communities played out in many situations where decisions for prescribing medicines and tests from outside market and referral to higher facilities were taken. Contrary to what we observed in the example above, we noticed a patient being referred to a higher facility twice and was asked to get an ultrasound done outside. The pregnant woman eventually delivered in a private hospital on the way to a medical college hospital.

*We were referred to the district hospital for delivery from our area by a doctor saying that the operation will be needed for delivery. At district hospital, we were not admitted and asked to go to medical college. On the way, my wife had severe labor pains, and we rushed to a private hospital in a hurry, where a normal delivery happened* (Beneficiary—2).

In other examples, an ideological view and belief of a manager in participatory management helped in bringing the community together to get donations for purchasing an ambulance for the hospital. Similarly, in one case, a nurse, because of her belief in the patient’s right to get services for free, resisted doctor’s prescription for branded medicines when generic medicines were available in the hospital.

### How did power struggles in actor interfaces affect JSSK implementation?

#### Strengthening of implementation and delivering policy promises

Some of the power dynamics helped to strengthen the implementation by facilitating decision making, streamlining of guidelines and procedures, fostered innovative solutions to local problems, improved demand from clients and subsequently ensured delivery of JSSK entitlements to many intended service users. At times, more powerful actors, because of their hierarchical positions clubbed with personal commitment and attitude, could drive the implementation process where it was stuck or moved slow. The authoritative power of such actors, when used constructively, allowed implementation decisions to happen less ambiguously. For instance, quick and clear decision making of one state officer led to the solution of persisting problems, which resulted in reduced complains of expenses made by beneficiaries.

*We had an officer, who soon after coming to office, cleaned up many ambiguities in JSSK implementation procedures. The state established a procurement cell, we received special drop back ambulances from the center, we expanded the JSSK list of medicines, our reporting and accounting improved. But we eventually persisted with same problems of ultrasound not being provided, medicines not available etc.… perhaps because vested interest and autonomy of district and facility staff is very high still and they don’t listen to NHM staff* (Middle manager*—*4).

Some actors used their autonomy and discretionary powers in a positive way and facilitated the implementation to champion many teething troubles. In the example above, a doctor (obstetrician 1), used professional ethics and sense of duty as well as a position of influence to ensure that the client gets free services. Similarly, collaborative interface engagements of actors at central, state, district and facility level helped to find solutions to major challenges such as availability of certain medicines in a medical college hospital and availability of ultrasound services in a district hospital.

*Understanding patients need for free ultrasonography, we allowed district and facility managers to do a tie-up with private. One hospital was successful in negotiating a price per case, which is very affordable to us. The same solution was offered to other districts, but they couldn’t do it* (Middle manager*—*2).

#### Constraining policy implementation and weakening of policy intent

While power dynamics in certain situations helped policy implementation, many practices of power led to a weakening of the policy intent and failure to deliver JSSK promises. The weakening of policy intent manifested through an overall fragmented implementation environment, a continuation of ambiguity in some procedures, inconsistent availability and irregular delivery of services, ignoring of or deviating from procedural guidance, not finding solutions for persisting problems, allowing practices of corruption and a gradual loss of interest as well as efforts to deliver JSSK promises.

The hesitation of state actors to originally implement JSSK, clubbed with an overall low system capacity to fulfil policy mandate was dominated over by a central push. This meant that scheme mostly started in a hurry and existed only on papers except for the patchy provision of some services. There was no infrastructure for providing free medicines, tests and ambulances and state was dependent on central funds for implementation. The state also had to fight the powers of the existing private market against delivering free services.

*Now we have an adequate number of drop back ambulances if you just count the number… But we still have people using personal vehicles to go back home. Why? …because most of them don’t get it. …. Why is this so? Because some places have more than the requirement and others, don’t get an ambulance when needed. Politicians interfere in our rational placing of ambulances* (Middle manager—3).

While these problems appear to be general systemic constraints, our findings show that actor relationships and their power dynamics contributed to many of these problems as highlighted in lifeworld analysis section above as well as in Table 5. In general, the practices of contestation and negotiations slowed down the implementation while resistance and ignoring policy mandate often led to non-delivery of services to clients. This can be cited with another example, which was seen in case of a client (beneficiary -2 above), who had to deliver the baby in a private hospital and incurred expenses about INR 10 000 (about US$1400), by the time they reached back home. The service providers, in this case, exercised their positional over and prevailing practice of referrals to refer the client from two government hospital citing the needs of higher-level medical care.

## Discussion

Implementation decisions and actions in JSSK experience were influenced by a complex interplay of practices of power which were underpinned by lived social realties or the *lifeworlds* of the actors. Everyday politics and power struggles of actors in this experience reinforced the idea that implementing health policies is not a function of merely having an implementation blueprint. Instead, it is influenced by the often neglected struggles of actors for resources, benefits, control, meanings, recognition and moral–ethical standings (Long, 2001). This builds on an actor centric understanding of policy implementation (Erasmus *et al.*, 2014; Gilson *et al.*, 2014; Sheikh *et al.*, 2014) and reinforces the notion that actors make meanings of their surrounding realities in relation to policy processes, and day to day decisions and actions are a function of actors’ power struggles (Long, 2001).

We noted that power was not always poised negatively as an influence on the implementation process but had positive effects as seen is some examples of clear decision making and collaborative uses of power to solve problems. Understanding positive and constructive uses of power in implementation can help in solving implementation challenges which are nested in power asymmetries in the health systems (Erasmus *et al.*, 2014; Sriram *et al.*, 2018; Gore and Parker, 2019). We found that implementation was facilitated strongly in examples where actors used their *lifeworld* experiences to deliver JSSK benefits to clients. On the contrary, a misalignment of lifeworld constructs with policy intent hampered the delivery of JSSK benefits to clients. Interpersonal relations and tensions over implementation processes slowed down the implementation process. As noted in the example of push from central actors to roll out the policy, the state actors and local private shop owners antagonized the policy intent, which constrained delivery of JSSK promises in early years. JSSK promises were further undermined by the prevailing interests and practices of doctors related to private prescriptions for medicines and tests. However, in some cases, higher functional autonomy of doctors, coupled with their professional ethics and moral sense of duty helped in the delivery of JSSK promises to patients.

**Figure 1:**
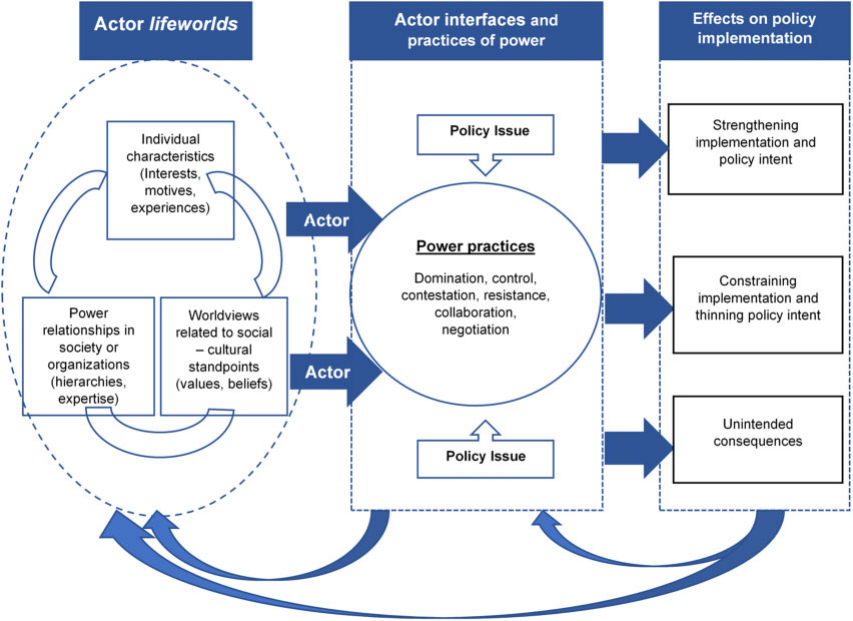
‘Interaction of actor lifeworlds’ and the ‘relationship of actor lifeworlds, actor interfaces and practices of power with their effects on health policy implementation’

Based on the findings of this study, we have depicted the relationships of the formation of actor interfaces and practices of power, a dynamicinteraction of three lifeworld categories and their effect on implementation in Figure 1.

Based on this experience, we argue that investment in understanding lived experiences of policy actors can enable researchers and policymakers, to find means for harnessing positive influences of power during policy implementation. Possibly, this can help in building systems which can harness the full potential of individual actors towards meeting policy and programme objectives. This is important because the power practices could exist in subtle manners and can be rooted beyond visible power structures, as observed in this study. These could be related to commonly neglected constructs like organizational culture and trust (Gilson *et al.*, 2014; Erasmus *et al.*, 2017), discretionary use of power by front line actors (Lipsky, 1980 ; Gilson, 2015) as well as agency, interests, norms, values and socio-cultural worldviews of actors (Sheikh and Porter, 2010; Hudson and Lefttwich, 2014). All of these may contribute to everyday struggles of actors over resources, meanings and control (Long and Liu, 2009) and shape the course of policy implementation.

While the broader implementation literature acknowledges the actor relationships and power as key influences on policy process (Walt and Gilson, 1994; Erasmus and Gilson, 2008; Erasmus *et al.*, 2014; Gilson *et al.*, 2014, 2018), there is a relative dearth of approaches to inform an analysis which considers everyday politics of actors and their power practices (Erasmus and Gilson, 2008; Sriram *et al.*, 2018). This experience informs that, as a complementary approach to commonly applied frameworks of power (Lipsky, 1980; Lukes, 2005; Gaventa, 2006; VeneKlasen and Miller, 2007), ‘actor interface analysis’ can offer nuanced steps of power analysis in health policy implementation. First, it provides an entry point to locate practices of power in interface of actors as observed in acts of domination, control, contestation, negotiation, consent, collaboration or resistance. Second, using *actor lifeworlds* as an analytical frame (Table 1), it can help to understand how lived realities of actors, which are embedded in their personal, organizational and social-cultural experiences manifest in the formation of actor interfaces, can lead to power struggles and can ultimately modify policy implementation.

### Limitations

One of the limitations of this analysis is that there could be multiple actor interfaces in an implementation experience, and all interfaces in JSSK experience could not be elicited in this paper. Second, the overall implementation of JSSK or any health policy for that matter is not shaped only by practices of power and many other influences matter. However, as the focus of this paper was to illustrate the role of practices of power on implementation, its scope remained limited to this aspect. Third, the paper focuses mostly on showcasing ‘how actor interface analysis was conducted’ and ‘how it can be used to explain power practices’. This which leaves out many discussions about how these learnings can be used for improving implementation given the scope of this paper and a permissible length.

## Conclusion

‘Actor interface analysis’ has been used in health policy implementation explicitly in two published studies only (Lehmann and Gilson, 2013; Barasa *et al.*, 2016) but can be useful to inform more implementation analyses. This paper, for the first time, outlines steps to use this approach in implementation analysis while offering a guiding framework (Table 1) to inform such analysis. However, this approach should be tested in more empirical studies for- further nuancing of ‘actor interfaces’ as a concept; detailing of ‘actor *lifeworlds*’ as a guiding framework for power analysis and to understand the influence of power struggles on implementation in varying contexts. While the day to day politics and power dynamics played out significantly in the implementation of JSSK, these cannot be seen in isolation. Such power practices were also a function of overall low capacity and readiness of the state health system to deliver free services, which were separately examined in this study.

## Supplementary Material

czaa125_Supplementary_File_1
