# Supplementary material for: Unpacking the implementation blackbox using 'actor interface analysis': how did actor relations and practices of power influence delivery of a free entitlement health policy in India?
Source: Health Policy Plan. 2020 Nov 6;35(Suppl 2):ii74–83. doi: 10.1093/heapol/czaa125 (PMC7646725; doi:10.1093/heapol/czaa125)
Supplement: czaa125_Supplementary_File_1 [file czaa125_supplementary_file_1.docx]

**Supplementary file 1**

**Guide for IDI with managers at different level (state/district/block/health facility)**

District/ Block :

Designation:

Place:

Date:

**(Informed consent would already have been received, before starting this part)**

different level of implementing actors) ?

*Note start time of the discussion -*

**Section 1: About the role and working context of the participant** (To understand working context of a person, their interest, motivation, values, approach to his/her work and life, general philosophies, etc.)

| 1. For how long have you been in this role? What are your overall responsibilities? | Let the respondent detail as much as possible. Encourage to speak more. |
| --- | --- |
| 1. What is your average day like? | Probe for routine activities a person does on the job , try to understand daily schedule |
| 1. How do you like/ enjoy your work? How do you like the overall work environment of your job? | Probe for - reasons to stick to this job, any shift in motivation/perception about quality of work life over the years or in respect to NHM , or in respect to any policies or professional growth or interpersonal / political dynamics |
| 1. What all administrative and financial powers do you have? Do you engage in problem solving of other managers like DPO, BMO or facility level staff like doctors, nurses or others? What all kind off issues come to you? How do you deal with such issues? | Probe for – understanding this managers idea about engagement with other staff for collaboration, problem solving, differences of opinion in general, management techniques, perception towards hierarchical rules etc.  Probe for any stories or examples of problem solving. |
| 1. How are you informed and engaged with a new policy/program from higher levels? And how do you engage higher level actors/ facility staff if you have to implement a new procedure/policy in your hospital? | Probe for – understanding information channels across different levels , to note the point of view of a person to approach his/her work |
| 1. How do you deal with or respond to dissent, resistance, noncompliance, good achievements etc in implementation of a policy? | Try to understand a person’s values towards his/her and others work, needs for task completion, the extent to which one would like to go, exercising their rights and interests |
| 1. How do you think is your area (according to level of manager – state/district/block/facility) is performing in context of delivering health services and implementing various programs/policies? | Probe for – History of health system and program capacities in this area, Historical performance, perception about temporal change in performance, perceived reasons for positive/negative shift including political reasons |

**Section 2: JSSK implementation and related dynamics** (To understand processes of JSSK implementation and related actor dynamics, politics and power negotiations related to the processes and their effect on implementation)

| 1. When and how did you get involved in JSSK ? 2. How did you hear about it, what was the process of involvement, who all were involved, what was your first perception/ initial thoughts about JSSK? Why do you think a program like JSSK was required or developed? Who was the in-charge of JSSK in this area before you? | Probes –  Perceived benefits/value of JSSK, perceived challenges with its arrival, any messages about policy, political agenda or demand from people |
| --- | --- |
| 1. What were the key activities planned under the program?   Or What was the planning to ensure free services for different components of JSSK – Free medicines, Free diagnostics, Free Food, Free transport, Free food, Exemption of all hospital charges like indoor stay, OPD fees, operation charges etc ?  Who were the key actors involved in this program or who were the stakeholders routinely interacted with? | Probe for –  All procedures/ processes laid down for ensuring free services across different entitlements of the scheme.  These probes culminate in the questions below. |
| 1. What is the current policy/procedure for ensuring free drugs under JSSK ? What were the planning and implementation steps for ensuring free drugs/medicines to beneficiaries in hospitals?   **Sub questions -**  What processes were developed for making drugs free? How was it different from earlier process? What were the steps used for implementing the new processes?  What was involvement of yourself and other actors at state/district/block/facility level? How did different actors like managers (state/district/block / facility), doctors, pharmacists etc respond to the new processes? What were the concerned raised by different actors and why? How were these addressed?  How was the response from outside chemists? Did they try to facilitate or interfere with the process?  What challenges were faced in implementing new drug processes? What worked better? How did implementation change over time - became better or worse or no effect? Any stories or examples?  What are beneficiaries or systems concerns/ challenges currently? What solutions have been discussed and by whom? | **Probe for –**  Difference in the old and new process after JSSK arrival, difficulties in the change, concerns of specific type of actor like – doctors, challenges related to human resource and their perception related concerns  Issue of drug quality or generic medicines, delay in purchase, stock out, outside chemist lobby, corruption in purchase procedures, doctor’s brand preference, previous prescription habits , unavailability of medicines for procedure/political reasons ? Instances of individual or group resistance or facilitation of a process? Any temporal change in practices / adherence and why?  Reasons for persisting challenges? Interpersonal dynamics / implementers’ discretion behind these reasons? |
| 1. What is the current process/ mechanism for ensuring free diagnostics? What were the implementation steps for ensuring free diagnostics (lab tests/ radiology – Ultrasound etc ) to beneficiaries in hospitals?   **Sub questions -**  What processes were developed for making diagnostic services free? How was it different from earlier process? What were the steps used for implementing the new processes?  What was involvement of yourself and other actors at state/district/block/facility level? How did different actors like managers (state/district/block / facility), doctors, pharmacists, lab personnel etc respond to the new processes? What were the concerned raised by different actors and why? How were these addressed?  How was the response from outside diagnostic shops? Did they try to facilitate or interfere with the process?  What challenges were faced in implementing new diagnostic service processes? What worked better? How did implementation change over time - became better or worse or no effect? Any stories or examples?  What are beneficiaries or systems concerns/ challenges currently? What solutions have been discussed and by whom?  What are the persisting challenges and why do you think these are there? What are the persisting challenges and why do you think these are there? What solutions have been discussed and by whom? | Probes - Map out all current processes for ensuring free diagnostics (Probe specially for Ultrasound services and non routine lab tests )  Difference in the old and new process after JSSK arrival, difficulties in implementing the change, concerns of specific type of actor like – doctors, challenges related to human resource and their perception related concerns  Availability of in-house 24*7 diagnostics, contract out arrangements, reimbursement to patients, outside shops lobby, corruption in purchase procedures, doctor’s preferences, beneficiary preferences for private services , instances of individual or group resistance or facilitation of a process?  Any temporal change in practices / adherence and why?  Reasons for persisting challenges? Interpersonal dynamics / implementers’ discretion behind these reasons? |
| 1. What is the current process/ mechanism for ensuring free transport services? What were the implementation steps for ensuring free transport (Engaging private ambulances, reimbursement for clients on private vehicles, New govt. ambulances) to beneficiaries / families coming to hospitals?   **Sub questions -**  What processes were developed for making transport free? How was it different from earlier process? What were the steps used for implementing the new processes?  What was involvement of yourself and other actors at state/district/block/facility level? How did different actors like managers (state/district/block / facility), doctors, pharmacists, lab personnel etc respond to the new processes? What were the concerned raised by different actors and why? How were these addressed?  What challenges were faced in implementing new free transport service processes? What worked better? How did implementation change over time - became better or worse or no effect? Any stories or examples?  What are beneficiaries or systems concerns/ challenges currently? What solutions have been discussed and by whom?  What are the persisting challenges and why do you think these are there? What are the persisting challenges and why do you think these are there? What solutions have been discussed and by whom? | Probes - Map out all current processes for ensuring free transport (Probe specially for type of ambulances used, their scope of work, payment procedures to ambulance companies, reimbursement process for clients on private vehicle use )  Difference in the old and new process after JSSK arrival, difficulties in implementing the change, challenges faced by any specific type of actors (ambulance drivers, ambulance company concerns, payment clerks ) and their perception related concerns  Unavailability of ambulances when a patient needs it like pick up from home or discharge or inter facility referral? Actors’ or procedure related reasons for unavailability? Beneficiary preferences for private services? Corruption/vested interests in ambulance contracts, delay in payments, instances of individual or group resistance or facilitation of a process? Any temporal change in practices / adherence and why?  Reasons for persisting challenges? Interpersonal dynamics / implementers’ discretion behind these reasons? Any process challenges? |
| 1. What is the current policy/procedure for providing free food to beneficiaries/ families under JSSK ? What were the implementation steps for ensuring free food to beneficiaries/ families?   **Sub questions -**  What processes were developed for making food free? How was it different from earlier process? What were the steps used for implementing the new processes?  What was involvement of yourself and other actors at state/district/block/facility level? How did different actors like managers (state/district/block / facility), doctors, pharmacists, lab personnel etc respond to the new processes? What were the concerned raised by different actors and why? How were these addressed?  What challenges were faced in implementing new free foodservice processes? What worked better? How did implementation change over time - became better or worse or no effect? Any stories or examples?  What are beneficiaries or systems concerns/ challenges currently? What solutions have been discussed and by whom?  What are the persisting challenges and why do you think these are there? What are the persisting challenges and why do you think these are there? What solutions have been discussed and by whom? | Probes- Map out all current processes for ensuring free food (in house kitchen/ outsourced/ reimbursement to families etc. If in house what processes? If outsourced what terms of contract? )  Difference in the old and new process after JSSK arrival, difficulties in implementing the change, challenges faced by any specific type of actors (facility managers, nurses, kitchen worker ) and their perception related concerns  Quality of food, round the clock availability, Beneficiary preferences for home food/ outside food? Delay in payments to kitchen workers/company, instances of individual or group resistance or facilitation of a process? Any temporal change in practices / adherence and why?  Reasons for persisting challenges? Interpersonal dynamics / implementers’ discretion behind these reasons? Any process challenges? |
| 1. What is the current policy/procedure for various hospital charges (user fees) to beneficiaries/ families under JSSK ? What were the implementation steps for ensuring that no hospital fees are charged from beneficiaries/ families?   What were the hospital charges related to JSSK services before this scheme? (Like OPD fees, indoor stay charges, food charges, operation charges, test charges etc )  At the start of JSSK, what procedures were identified for exempting all charges in hospitals? How were these different from earlier procedures? Who were the stakeholders involved in ensuring no charges for hospital services?  What challenges were faced in implementing new use fee exemption processes? What worked better? How did implementation change over time - became better or worse or no effect? Any stories or examples?  What are beneficiaries or systems concerns/ challenges currently? What solutions have been discussed and by whom? | Probe – Procedures laid down for exempting user charges in hospital and steps to make them happen  Reasons for ongoing payments after JSSK arrival, Rogi Kalyan Samiti (hospital committee) related issues? Informal / formal tips to service providers? Payments asked by service providers? |
| 1. How communities were made aware of JSSK entitlements?   What was the plan made initially for rising awareness about JSSK entitlements in communities?  How were these plans implemented or what steps were actually taken to raise awareness?  How did communities react to the scheme? What was the opinion of hospital staff/doctors/managers about JSSK when it arrived?  How doctors did facilitated raising JSSK awareness? What was the response of outside chemist, diagnostic providers? Was there any resistance from any type of service providers or from any level initially?  How do you think is the awareness about all entitlements of JSSK in public? Which services need more awareness? What more do you think can be done? | Do you think there is an effect of community demand/ awareness on delivery of free services? |
| 1. What happens when an intended service is not available or cannot be delivered because of some bottleneck? | Probe for what kind of bottlenecks are faced and how are they resolved? (probe to get some examples and stories of an issue which was handled) |
| 1. What do you think doctors or nurses in health facilities think about free services? How was there response when scheme was launched? | Probe for – any group lobbies, any individual enthusiasm or resistance , interpersonal difference of opinion about policy entitlements or implementation idea between any two actors or group of actors , any specific demands of doctors / nurses or other groups . Any stories or examples? |
| 1. How do you think your area (state/district/block/facility) is doing in terms of JSSK implementation? | Probe for perceived performance on JSSK, probe for citing variations within this managers’ area for JSSK performance (like between two blocks in district) and perceived reasons for this difference |
| 1. From your experience, what are the factors which would ensure better implementation of JSSK activities and delivery of entitlements? | Probe for any perceived need of- change in policy entitlements? Need of better implementation steps and processes? Relationship management with various policy actors? Engagement with community? Need of better coordination / communication across levels? Or anything else? |

*Note end time of the discussion*
